# Comparison of natural ostiodilatation and endoscopic sinus surgery in the same patient with chronic sinusitis^☆^

**DOI:** 10.1016/j.bjorl.2018.09.006

**Published:** 2018-10-22

**Authors:** Ahmet Kutluhan, Hüseyin Çetin, Hayati Kale, Özmen Kara, Halil İbrahim Mişe, Tolga Oğuzhan, Kadir Şinasi Bulut

**Affiliations:** a Yildirim Beyazit University, Department of Otorhinolaryngology, Ankara, Turkey; b Ataturk Education and Research Hospital, Department of Otorhinolaryngology, Ankara, Turkey; c Dr. Sami Ulus Education and Research Hospital, Department of Otorhinolaryngology, Ankara, Turkey; d Dr Nafiz Körfez Sincan State Hospital, Department of Otorhinolaryngology, Ankara, Turkey; e Kackar State Hospital, Department of Otorhinolaryngology, Rize, Turkey; f Malazgirt State Hospital, Department of Otorhinolaryngology, Muş, Turkey

**Keywords:** Balloon sinoplasty, Endoscopic sinus surgery, Chronic sinustis, Natural ostiodilatation, Sinuplastia com balão, Cirurgia endoscópica nasossinusal, Rinossinusite crônica, Dilatação do óstio natural

## Abstract

**Introduction:**

Chronic rhinosinusitis is a broad clinical syndrome characterized by mucosal inflammation of the nose and paranasal sinuses. In order for the paranasal sinuses to maintain their physiological functions; the ostiomeatal complex drainage pathways must be open. Surgical procedures are an important treatment option in patients who do not respond adequately to medical treatment. Although the methods and instruments used in functional endoscopic sinus surgery have continued to improve in recent years, the scar tissue formed during operation disrupts the drainage of the sinuses and reduces postoperative success. The natural ostiodilatation method, which is performed by balloon sinoplasty method, has become more and more popular in recent years.

**Objectives:**

To compare the technique of balloon sinoplasty with the classical functional endoscopic sinus surgery method by considering the severity of chronic sinusitis on the same patient.

**Methods:**

Total of 61 chronic sinusitis patients was included in the study. Paranasal sinus tomography of the patients was taken and according to the Lund–Mackay scoring, chronic sinusitis levels were determined. Cases were divided into two groups: Group 1 (severe chronic sinusitis group) and Group 2 (mild chronic sinusitis).

**Results:**

There was no statistically significant difference in the results of comparison of sinuses which underwent balloon sinoplasty and classical functional endoscopic sinus surgery in Group 2 after Lund–Mackay scores. However in Group 1, the results of the comparison of postoperative Lund–Mackay scores of the balloon sinoplasty and the classical endoscopic operation were statistically significantly lower than those of the face half operated with the classical functional endoscopic sinus surgery.

**Conclusion:**

The success of balloon sinoplasty in patients with mild sinusitis is the same as in classic functional endoscopic sinus surgery. However, as the severity of sinusitis increases, the efficacy of balloon sinoplasty decreases.

## Resumo

**Introdução:**

A rinossinusite crônica é uma síndrome clínica ampla, caracterizada por inflamação da mucosa nasal e seios paranasais. Para que os seios paranasais mantenham suas funções fisiológicas, as vias de drenagem do complexo ostiomeatal devem estar abertas. Os procedimentos cirúrgicos são uma importante opção de tratamento em pacientes que não respondem adequadamente ao tratamento clínico. Embora os métodos e instrumentos utilizados na cirurgia endoscópica funcional dos seios paranasais tenham melhorado continuamente, o tecido cicatricial formado durante a cirurgia interrompe a drenagem dos seios nasais e reduz o sucesso pós-operatório. O método natural de dilatação ostial, que é aplicado por meio da técnica de sinuplastia com balão, tem se tornado cada vez mais popular nos últimos anos.

**Objetivos:**

Comparar a técnica de sinuplastia com balão com o método convencional de cirurgia endoscópica, no mesmo paciente, considerando a gravidade da rinossinusite crônica.

**Método:**

Foram incluídos no estudo 61 pacientes com rinossinusite crônica. Por meio de tomografia dos seios paranasais e de acordo a escala de Lund-Mackay foram determinados os graus da rinossinusite crônica. Os casos foram divididos em dois grupos: Grupo 1 (grupo com rinossinusite crônica grave) e Grupo 2 (rinossinusite crônica leve).

**Resultados:**

No Grupo 2 não houve diferença estatisticamente significante, segundo a escala de Lund-Mackay no resultado da comparação dos seios paranasais nos quais foram realizadas a sinuplastia com balão e por cirurgia endoscópica convencional. No entanto, no Grupo 1, os resultados da comparação dos escores pós-operatórios de Lund-Mackay mostraram-se estatística e significativamente melhores naqueles submetidos à cirurgia endoscópica funcional dos seios paranasais.

**Conclusão:**

O sucesso da sinuplastia com balão em pacientes com rinossinusite leve é o mesmo da cirurgia endoscópica funcional dos seios da face tradicional. No entanto, à medida que a gravidade da rinossinusite aumenta, a eficácia da sinuplastia com balão diminui.

## Introduction

Chronic rhinosinusitis (CRS) is a broad clinical syndrome characterized by mucosal inflammation of the nose and paranasal sinuses. The most important pathologic factor in the development of CRS symptoms is the occurrence of a blockage state that prevents the physiological drainage of the ostiomeatal complex (OMC). Major factors that contribute to the deterioration of this physiological drainage in recent years are anatomical variations, allergens, infectious agents and air contaminants causing nasal epithelial inflammation.^1^

The primary treatment of CRS is medical treatment, which provides remission in many patients.^2^ Surgical procedures are an important treatment option in patients who do not respond adequately to medical treatment, have obstructive anatomical variations of sinus drainage in imaging and examination results, develop sinusitis complications and have allergic fungal infection.^3^ The basic principles of functional endoscopic sinus surgery (FESS) are based on endoscopic visualization of the nose by an endonasal approach and removal of the drainage-inhibiting tissues using special instruments.^4^ Although the methods and instruments used in FESS surgery have continued to improve in recent years, the scar tissue formed during operation disrupts the drainage of the sinuses and reduces postoperative success. Minimally invasive methods have been developed to further reduce scar formation; methods are needed to provide natural dilatation of the sinus ostia.

The natural ostiodilatation method, which is performed by the balloon sinoplasty method, has become more and more popular in recent years. This method provides a faster detection of localization of the sinuses, especially the frontal sinus, and shortens the duration of the operation.^5^ Ostiodilatation procedures performed by balloon-sinoplasty methods cause less damage to surrounding tissues than conventional FESS methods.^6^, ^7^ For this reason, scar tissue develops less frequently after balloon sinoplasty, and the possibility of recurrent obstruction of the sinus drainage path is severely reduced.

Although there are many studies regarding the efficacy of balloon sinoplasty, there is no previous study in the literature investigating the efficacy of balloon sinoplasty according to the severity of chronic sinusitis. We compared the technique of balloon sinoplasty with the classical FESS method by considering the severity of chronic sinusitis on the same patient in this study. Thus, we tried to minimize the factors related to the patient.

## Methods

A total of 61 chronic sinusitis patients (33 males, 28females, aged 23 ± 45 years, mean age 34.4 years) who were evaluated at our clinic between 2015 ± 2016 were included in the study. The study was approved by the ethics committee (Approval number: 26379996/186) and an informed consent form was signed. Inclusion criteria: being between the ages of 18 ± 65; have not previously had chronic sinusitis surgery; not having cystic fibrosis, ciliary dyskinesia; and not having a disease that would disrupt wound healing, such as diabetes or hypertension. According to the Lund–Mackay scoring, asymmetric sinusitis severities (two and above difference) in the right and left half of the faces were excluded from the study.^8^

Daily methyl-prednisolone treatment was given preoperatively orally to each patient for 5 days at a dose of 1 mg/kg (min: 40 mg; max: 100 mg). Paranasal sinus tomography of the patients was taken and interpreted by the same radiologist. According to the Lund–Mackay scoring, chronic sinusitis levels were determined by 12 points (Table 1).

**Table 1:** Lund–Mackay scoring.

	Right-sided sinuses	Left-sided sinuses
Maxillary (0, 1, 2)		
Anterior ethmoid (0, 1, 2)		
Posterior ethmoid (0,1,2)		
Sphenoid (0, 1, 2)		
Frontal (0, 1, 2)		
Ostiomeatal complex (0, 2)^a^		

No abnormality = 0; Partial opacification = 1; Total opacification = 2.

a Not obstructed = 0; obstructed = 2.

Cases were divided into two groups: Group 1: Patients with Lund–Mackay Score of 7 and above (severe chronic sinusitis); Group 2: Patients with Lund–Mackay Score of 6 and below (mild chronic sinusitis).

Patients with the same (mild/mild, severe/severe) chronic sinusitis level according to the Lund–Mackay scoring scale were selected for right and left half.

Surgery: all procedures carried out were performed under general anesthesia. Balloon sinoplasty system consisted of relieve catheter, guide catheters and cables, transillumination light source and pressure gauge balloon inflator apparatus.

A classical FESS procedure was carried out on the sinuses of a randomly selected half of the patients’ face. Initially, all the polyps in the nasal passages were cleaned. The maxillary sinus ostium was identified after uncinectomy and enlarged in the postero-inferior direction with the aid of cutting forceps. Anterior and posterior ethmoidectomy was performed. Before reaching the frontal sinus ostium, mucosal thickening and cells that obstructed the frontal recess were excised. Later, cells and tissues that obstructed the frontal ostium were excised with the aid of the J curette. When necessary soft tissues around the sphenoid sinus ostium were removed and the sinus ostium expanded inferiorly. The upper concha lower half was excised for this operation.

Balloon sinoplasty was performed on the other half of the face. Before the procedure was initiated, simple polypectomy was performed by excising polyps in the nasal cavity, if present. The balloon transillumination light source was then guided through the nasal cavity and verified through the skin that it was inserted into the desired sinus. The ostia of maxillary, anterior–posterior ethmoid, frontal and sphenoid sinuses were identified and dilated with balloon catheter. Balloon dilatation was performed to the ethmoid sinus with the technique described in a previous study.^7^ The balloon catheter was placed in the ostium of the sinuses and inflated to a pressure of 12 bar and held at this level for about 10 s. Then the balloon was deflated and removed. After ostium dilatation, if there was secretion within the sinus, aspiration with straight or curved aspirator was carried out. Any bleeding was controlled with sponges. Patients were discharged on the first day postoperatively, and one week of antibiotic treatment and pressure nasal wash solutions were prescribed. Patients were checked in the first week postoperatively and aspirated intranasally. Patients who were called to follow-up between 13 and 17 months after surgery again had their paranasal sinus tomography taken. These tomography images were also evaluated by the radiologist who read them before the operation. Lund–Mackay scoring was performed and Group 1 and Group 2 were compared within themselves. The operations performed on the sinuses of the patients are summarized (Table 2).

**Table 2:** Procedures performed in classical FESS and balloon sinoplasty.

	FESS	Balloon sinoplasty
Maxillary sinus	Unsynectomy + resection of ostium posteroinferioris	Maxillary ostium balloon dilatation
Anterior ethmoid sinus	Anterior ethmoidectomy	Ethmoid bulla perforation and balloon dilatation
Posterior ethmoid sinus	Posterior ethmoidectomy	Ethmoid bulla perforation and balloon dilatation
Frontal sinus	Frontal recess and ostium expansion	Baloon dilatation of frontal recess and ostium
Sphenoid sinus	Expansion of sinus ostium inferiorly	Baloon dilatation of sphenoid sinus ostium

The statistical analysis was carried out with SPSS v. 23.0 for Windows (SPSS, Chicago, IL) and statistical significance was determined at *p* < 0.05. Student's *t* test was used to evaluate Lund–Mackay score averages of sinuses. Chi-square test was used to evaluate gender variable and Student's *t* test was used to evaluate age variable among groups.

## Results

There was no statistically significant difference between Groups 1 and 2 in terms of demographic characteristics (Table 3).

**Table 3:** Demographic characteristics.

	Group 1 (*n* = 34)	Group 2 (*n* = 27)	*p*
*Age*	33.4 ± 5.6	35.7 ± 6.1	0.13^a^

*Gender*			0.83^b^
Male	18 (52.9%)	15 (55.6%)	
Female	16 (47.1%)	12 (44. %)	

a Student's *t* test.

b Pearson Chi-square test.

Pre and post-operative Lund–Mackay scores of all patients are shown in Table 4.

**Table 4:** Preoperative and postoperative Lund–Mackay scoring of patients in Group 1 and 2.

Group 1				Group 2			
Balloon sinoplasty side		Classical FESS side		Balloon sinoplasty side		Classical FESS side	
Pre-op	Post-op	Pre-op	Post-op	Pre-op	Post-op	Pre-op	Post-op
9	6	9	4	5	3	6	6
11	7	11	5	6	4	6	0
8	5	7	5	4	3	4	2
12	6	11	4	4	4	4	5
7	5	7	5	3	3	3	2
11	4	11	7	6	5	6	0
9	5	10	4	6	4	6	3
7	8	8	5	5	7	5	4
7	6	7	5	6	5	6	0
8	9	7	5	6	3	6	6
12	5	12	5	6	4	6	2
10	7	11	6	5	2	5	4
12	4	12	3	6	1	6	3
12	3	12	4	5	1	5	3
9	5	9	4	5	3	5	3
8	4	8	3	4	5	4	3
11	5	10	2	6	0	6	2
10	6	10	3	6	2	6	0
11	3	10	6	5	0	5	2
9	7	9	5	6	3	6	3
7	5	7	7	4	0	4	2
7	8	8	9	4	4	4	6
7	6	7	6	3	5	3	2
9	9	9	3	6	3	6	5
12	5	12	5	6	2	6	6
10	4	9	9	4	1	4	7
10	5	10	4	5	2	5	5
11	8	11	5				
12	7	12	5				
10	5	10	3				
8	6	8	3				
9	5	9	2				
12	4	12	5				
9	7	8	4				

There was no statistically significant difference between the results of the comparison of the Lund–Mackay scores of the sinus treated by balloon sinoplasty and the classical FESS among all patient, without grouping patients as mild or severe (Table 5).

**Table 5:** Comparisons of balloon sinoplasty and classical FESS scores across all patients.

	*n*	Mean	Std. deviation	*p*
Balloon	61	4.47	2.23	0.24^a^
FESS	61	4.03	1.96	

a Student's *t* test.

**Figure 1:**
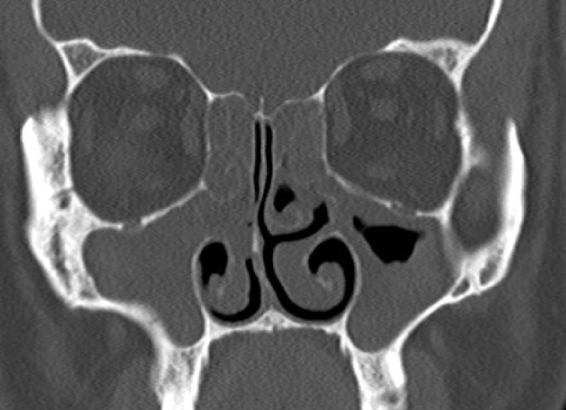
A pre-operative coronal paranasal tomography section of a case in Group 2.

**Figure 2:**
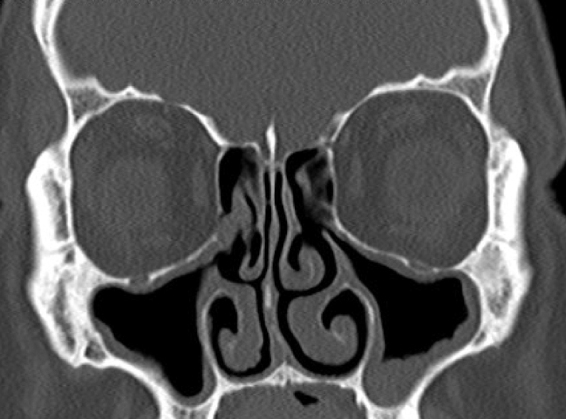
The right half of the case in Fig. 1, one year tomography after performing baloon sinoplasty to the right sinuses and classic FESS operation to the left sinuses.

There was no statistically significant difference in the results of comparison of sinuses undergoing balloon sinoplasty and classical FESS in Group 2 after Lund–Mackay scores (Table 6) (Figure 1, Figure 2).

**Table 6:** Comparisons of balloon sinoplasty and classical FESS scores in Group 2.

	*n*	Mean	Std. deviation	*p*
Balloon	27	2.92	1.97	0.63^a^
FESS	27	3.18	2.03	

a Student's *t* test.

In Group 1, the results of the comparison of postoperative Lund–Mackay scores of the balloon sinoplasty and the classical FESS operation were statistically significantly lower than those of the face half operated with the classical FESS (Table 7).

**Table 7:** Comparisons of balloon sinoplasty and classical FESS scores in Group 1.

	*n*	Mean	Std. deviation	*p*
Balloon	34	5.70	1.56	0.01^a^
FESS	34	4.70	1.64	

a Student's *t* test.

**Figure 3:**
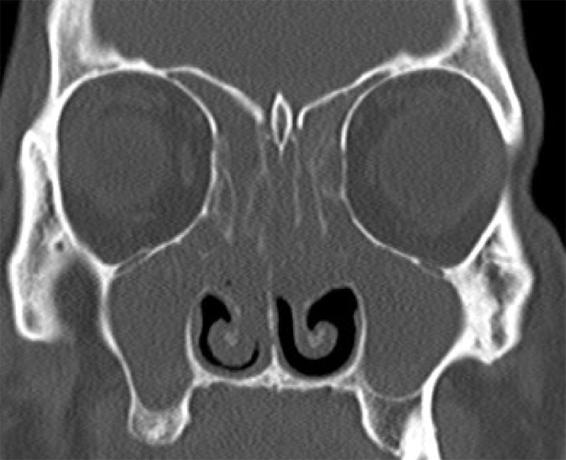
Preoperative paranasal tomography of a patient with exacerbated sinusitis.

**Figure 4:**
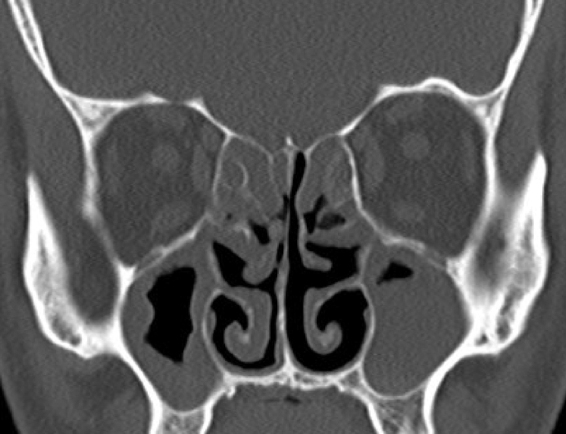
The right half of the case in Fig. 3, one year tomography after performing baloon sinoplasty to the right sinuses and classic FESS operation to the left sinuses.

Postoperatively, in some cases, the symptoms of sinusitis became more severe during follow-up visits according to both anamnesis and examination findings. When the postoperative CT scans were examined, more severe or a similar sinusitis table that was similar to the preoperative condition was seen in both the balloon and the classical FESS surgeries. Also in the radiological examination of these patients, the balloon and the classical FESS were not different from each other in terms of sinusitis recurrence (Figure 3, Figure 4).

## Discussion

Although the etiology of CRS with or without polyposis is not fully understood, the deterioration of sinusoidal drainage is considered to be one of the most important factors today.^9^, ^10^ Therefore, surgical procedures used in the treatment of chronic sinusitis are aimed at opening the drainage of the sinuses. However, in classical methods, the surrounding tissues are damaged when attempting to remove the obstruction at the level of the sinus ostia. Mucosal destruction occurs around the ostium when drainage of other sinuses, such as excision of the uncinate protrusion, occurs when the maxillary ostium is opened. Scar tissue that may result in this destruction may cause the drainage to diminished by closure of the sinus ostium. Many studies have reported recurrences up to 10% after sinus surgery, depending on the technique used.^11^, ^12^ Many patients may have to be re-operated due to these recurrences and this can have negative effects on both patient morbidity and the country's economy. In the balloon sinoplasty method, a natural ostiodilatation favors lessi damage to the surrounding tissues of the sinus ostium. Thus less scarring is ensured.

Levine et al. reported a study conducted with more than a thousand patients 69.5% were treated with the balloon sinoplasty method.^13^ They found this rate as 16.7% in the treatment with classical FESS methods. Again, in the treatment of frontal sinus, the rate of improvement of balloon sinoplasty method was superior with the rate of 62.2% compared to classical FESS methods as 2.4%.

Although classical FESS methods have been shown to reduce morbidity, it has been shown that major complications such as optic nerve damage, cerebrospinal fluid fistula, and major bleeding can occur in 1.1%. It has also been shown that it may lead to minor complications such as infection, minor bleeding, periorbital emphysema, synechiae.^5^, ^14^, ^15^ Compared to conventional FESS surgery, the risk of complications is very low for minimally invasive methods such as balloon-sinoplasty, since there is no portion of this method that would damage the surrounding mucosa and bone structure other than controlled balloon dilatation.

There are many studies in the literature investigating the role of balloon sinoplasty in chronic sinusitis surgery.^16^, ^17^, ^18^ No grouping was performed according to the severity of chronic sinusitis in any of these studies. In the analysis without grouping patients, we also observed that the results of conventional FESS and balloon sinoplasty did not have superiority to each other. But, when we divided the cases into mild and severe cases, we observed that the classical FESS method was superior in the group with severe sinusitis (Group 1); and that the methods are equally effective in mild sinusitis (Group 2). That shows pre-operative evaluation of sinusitis severity is an important factor in choosing the operation method. This result is a noticeable outcome of this study on balloon sinoplasty.

In a previous study conducted with 10 cases, one face of the patients underwent a classic FESS, while the other half had sinus balloon sinoplasty, as in this study.^7^ When preoperative and postoperative tomography of all cases were evaluated by Lund–Mackay scoring, it was seen that balloon sinoplasty and classical FESS-operated sinuses were not superior to each other in terms of healing, although 10 patients of that study complied with Group 1 in this study. In this study, we reached a statistically significant difference in favor of FESS. This may be due to the fact that the number of patients in this study is higher.

Postoperatively, sinusitis findings were more severe in some cases in total. The same severe recurrence of both balloon and FESS side on the radiological examination suggests that there may be no significant difference between the two methods in terms of recurrence. However, there is a need for a study to be conducted with a larger number of cases for a comparison between the two methods in terms of recurrence.

## Conclusion

The success of balloon sinoplasty in patients with mild sinusitis is the same as in classic FESS. However, as the severity of sinusitis increases, the efficacy of balloon sinoplasty decreases. Classic FESS surgery still seems to be a more effective method in cases with severe sinusitis. However balloon sinoplasty can be preferred because of less risk of complication.

## Conflicts of interest

The authors declare no conflicts of interest.
